# A Study on Laser Enhanced Electrodeposition for Preparation Fe-Ni Alloy

**DOI:** 10.3390/ma13163560

**Published:** 2020-08-12

**Authors:** Zhaoyang Zhang, Yucheng Wu, Anbin Wang, Kun Xu, Xueren Dai, Hao Zhu, Shuai Yang

**Affiliations:** Laser Technology Institute, School of Mechanical Engineering, Jiangsu University, Zhenjiang 212000, China; 2211803062@stmail.ujs.edu.cn (Y.W.); 2211803060@stmail.ujs.edu.cn (A.W.); xukun@ujs.edu.cn (K.X.); 2111703013@stmail.ujs.edu.cn (X.D.); haozhu@ujs.edu.cn (H.Z.); 2211803033@stmail.ujs.edu.cn (S.Y.)

**Keywords:** laser energy, electrodeposition, Fe-Ni alloy, properties

## Abstract

In this paper, a method of laser enhanced electrodeposition is used for preparation of Fe-Ni alloy, which exhibits a significant advantage in fabrication of alloys. The effect of laser energy on Fe-Ni alloy electrodeposition by the manner of reciprocating scanning is studied. Results show that laser irradiation can improve the surface morphology, micro-structure and mechanical properties of Fe-Ni alloy. The results are useful for the development of a new method to synthesize Fe-Ni alloy with better properties.

## 1. Introduction

Excellent performance such as low thermal expansion, high corrosion resistance and hardness makes Fe-Ni alloy widely used in the industrial field [1,2,3]. Electrodeposition is a normal method to produce Fe-Ni alloy, however, accompany with some defects such as pores and large internal stress [4,5,6,7]. Laser is widely used in surface engineering technology and special processing because of high energy density, directionality and monochromaticity [8,9,10]. Laser irradiation can change the electrode state and generate photoelectrochemical effect, thermal-electrochemical effect and mechanoelectrochemical effect, which is beneficial for the processing of electrodeposition [11,12].

In past few decades, some good results of laser enhanced electrodeposition have been achieved by many scholars. I. Zouari et al. [13] added a pulsed laser to spray electrodeposited zinc and found that it is possible to refine grains and improve the morphology of the coating. C. H. Cho et al. [14] achieved selective copper deposition on a maskless metal surface by combining laser and electrodeposition techniques. T. Yan et al. [15] found that introducing laser into electrodeposition can reduce the residual stress. Z. L. Yu et al. [16,17] found that laser irradiation can affect the growth direction and grain size of ionic liquid electrodeposition process. S. H. Chen et al. [18] achieved successfully the copper micro/nanoparticles by using laser-enhanced electroplating and found the laser could reduce the particle size. However, the method of laser enhanced electrodeposition to fabricate alloy is rarely reported, especially the Fe-Ni alloy.

In this paper, a laser electrochemical compound processing system was conducted and the laser thermal effect on preparation of Fe-Ni alloy by the manner of reciprocating scanning in electrodeposition was investigated. Results of laser thermal effect on the morphology, micro-structure and mechanical properties of Fe-Ni alloy was discussed.

## 2. Experimental Details

In this study, a laser electrochemical composite machining device were conducted, including laser irradiation system and electrodeposition machining system. Laser energy was introduced at single pulse energy of 10 μJ, 12.5 μJ, 15.0 μJ, 17.5 μJ, 20.0 μJ by using a picosecond laser (Edge Wave, Aachen, Germany). In this experiment, the frequency is 1 MHz, the defocus is 1mm, the scanning speed is 3000 mm/s and the line spacing of the scan path is 20 μm. In addition, electrodeposition machining system mainly includes a power supply (GKPT, Shenzhen, China) and the duty cycle is 50%, the frequency is 1000 Hz, and the current density is 5 A/dm^2^ in this experiment. A stainless-steel sample (10 mm × 10 mm × 1.5 mm) was used as a cathode, and a pure Fe sheet and an electrolytic Ni sheet inserted in anode bags were used as anode. The samples were mechanically polished with abrasive papers from 400 to 2000 grades and then were ultrasonically cleaned for 10 min before plating. The details of electrolyte composition and plating conditions are presented in Table 1.

The surface morphology was measured using scanning electron microscopy (Hitachi S-3400) (HITACHI Corp, Tokyo, Japan) and the chemical composition was observed by using energy dispersive X-ray spectroscopy (EDS) (HITACHI Corp, Tokyo, Japan) analysis. The value of roughness of surface was measured using laser scanning confocal microscope (KEYENCE VK-X200 series) (KEYENCE Corp, Shanghai, China). The phases of the coatings and grain size determination were characterized with X-ray diffraction (XRD) technique using Cu Ka radiation (BRUKER/D8ADVANCED Diffractometer, 30 kV, 30 mA) (BRUKER Corp, Karlsruhe, Germany) and the results were interpreted by X-pert High Score software (Version 3.0, PANalytical B.V. Corp, Almelo, Netherlands). Also, the grain size was calculated by using Scherrer Equation (1) [19]:

(1) $$D=\frac{0.89\lambda }{\beta \cos\theta }$$

where D is the crystallite size, λ is the incident radiation (1.5418 Å), β is the corrected peak width at half-maximum intensity and θ is the angular position. The scan was performed from 2θ angles of 20–80° with a step size about 5°/min.

The micro-hardness tests were conducted on the surface of the coatings using a FM-ARS900 micro-hardness tester (Xinci Corp, Shanghai, China) with a load of 100 g applied for 15 s. The internal stress tests were conducted by using X-350A X-ray stress analyzer (Aisite Corp, Handan, China). Corrosion behavior were evaluated in 3.5% NaCl solution for seven days by measuring the quality change.

**Figure 1:**
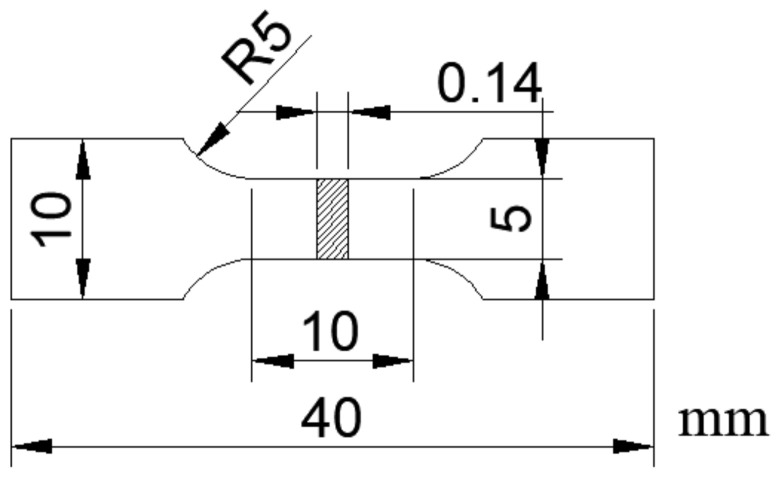
Dimensions of the dumbbell-type specimens used for the tensile tests.

The specimens used for the tensile test were fabricated the dumbbell type as shown in Figure 1. The tensile tests were conducted using an Instron-type universal tester (Autograph AG-250kN, Shimadzu Corp, Kyoto, Japan) under a tensile speed of 0.1 mm/min.

## 3. Results and Discussions

### 3.1. Surface Morphology and Chemical Composition

**Figure 2:**
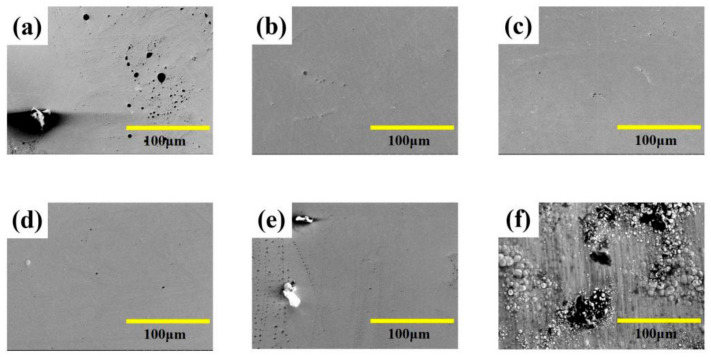
SEM micro-graphs of coatings with varying laser energies at: (**a**) 0 μJ, (**b**) 10.0 μJ, (**c**) 12.5 μJ, (**d**) 15.0 μJ, (**e**) 17.5 μJ, (**f**) 20.0 μJ.

**Figure 3:**
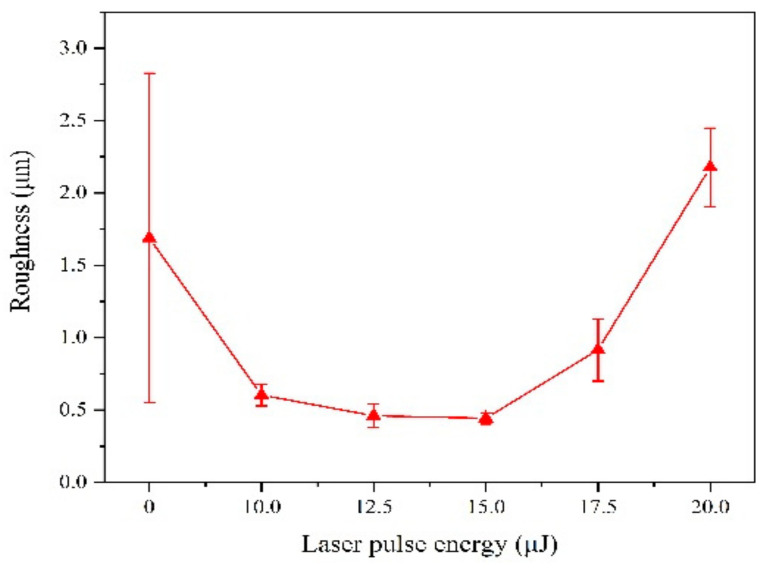
Surface roughness of coatings with varying laser energies.

Surface morphology of Fe-Ni alloy was observed from Figure 2 under different laser single pulse energy. The coatings have a mixture morphology accompany with small needles and air hole without laser irradiation, while the surface becomes uniform and dense at the lower laser energy (10.0 μJ–15.0 μJ) from Figure 2a–d. The absorption of laser heat by ions reduces the activation energy, so that the laser can make the reduction easy. Therefore, the nucleation rate is greater than the growth rate [11], which is contribute to grain refinement. Besides, the laser irradiation suppresses the adhesion of impurities to the substrate, so that the granular protrusions were reduced. In addition, the laser irradiation area can form micro-area agitation due to temperature gradient, which can remove bubbles in time [4,11,20]. However, according to Figure 2e,f, the coatings were rough with big bumps and holes morphology. This is because, when the laser single pulse energy becomes larger, the hydrogen evolution phenomenon is intensified, which affects the laser action and causes some defects such as pits, pocking and cracks. When the laser single pulse energy goes further, the integrity of the coating surface will be destroyed. The surface roughness of Fe-Ni alloy was shown in Figure 3, which is consistent with surface morphology.

**Figure 4:**
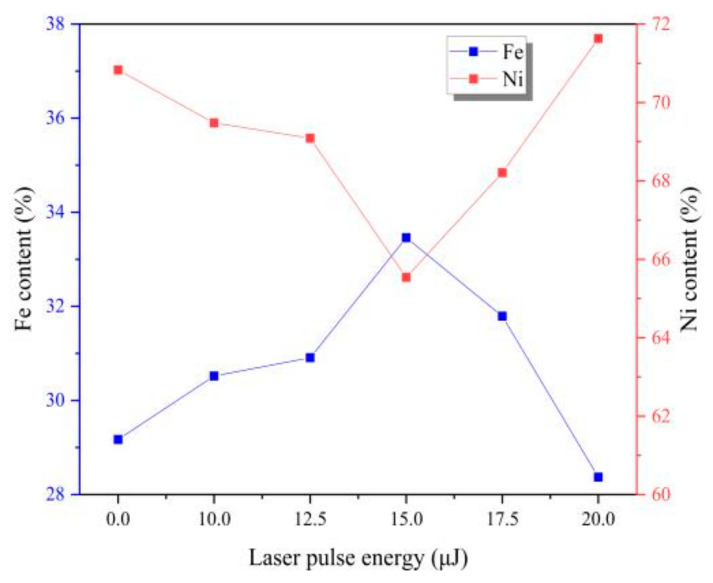
Chemical composition of coatings with varying laser energies.

In fact, Fe-Ni alloy coatings have the abnormal co-deposition behavior. Deposition of Fe is mainly controlled by diffusion process, while the deposition of Ni is mainly controlled by activation [4]. It can be seen from Figure 4 that the content of Fe element firstly increases and then decreases with the increase of laser energy. When adding laser irradiation, the catholic over-potential is enhanced which leads to increase the activation of reactions [4,11,20], which is beneficial for the deposition of Ni. The laser thermal effect would cause obvious turbulence in the surface of cathode and electrolyte, which could form the micro-region stirring and promote the effect of diffusion process [4,11,20]. According to the change of element content, we judge that when introducing the laser irradiation, the effect of laser thermal effect on diffusion is greater than that of activation comparing to without laser irradiation. So, we can conclude that the influence of laser energy on Fe is more obvious than it is on Ni in this experiment.

### 3.2. Structure Characterization and Internal Stress

**Figure 5:**
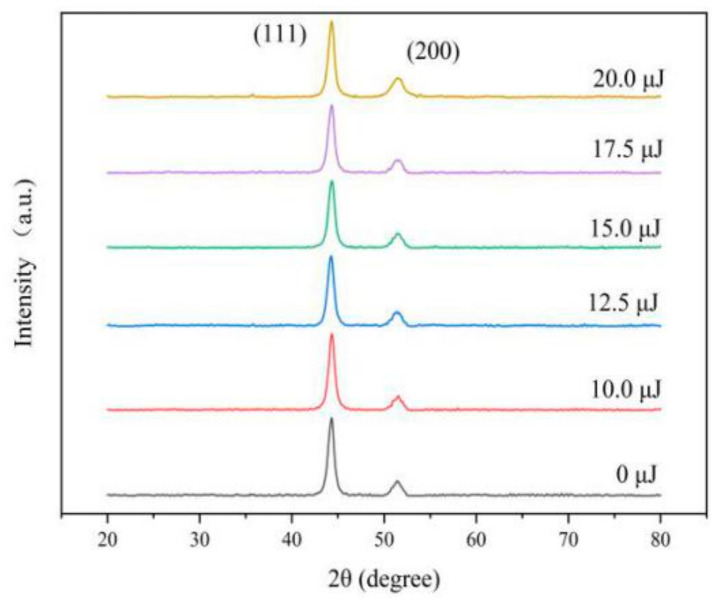
XRD pattern of coatings with varying laser energies.

**Figure 6:**
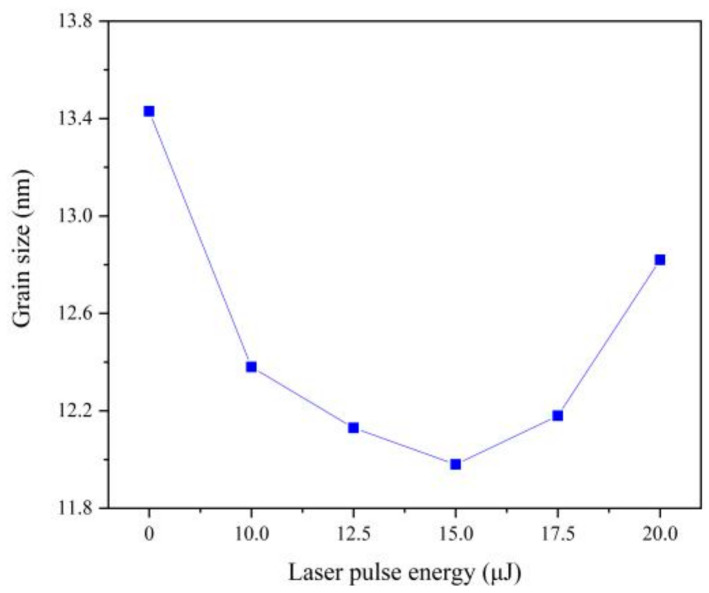
Grain size of coatings with varying laser energies.

The XRD peaks appear near 44° and 51° in 2θ from Figure 5, which is a typical surface crystal structure and is consistent with the (111) and (200) planes of the face centered cubic of Ni. In addition, the XRD peaks have few differences of varying laser energies, indicating that the laser did not change the crystal structure of Fe-Ni alloy.

(111) plane is the preferred growth plane of Fe-Ni alloy. So, the grain size was calculated by Scherrer equation according to the diffraction intensity of (111) plane, which decreases with the increasing of laser energy, as can be seen from Figure 6. The nucleation rate of electrodeposition increases exponentially with increasing over-potential [20]. According to Nernst equation [11], the over-potential was increased by adding laser thermal effect, which can promote the nucleation rate and reduce the grain size. Besides, with the increase of Fe content, the lattice distortion will be aggravated, resulting in lattice defects such as dislocation and vacancy. When the number of dislocations reaches a certain degree, small-angle grain boundaries will appear, leading to the refinement of grain size [2,21]. When the single pulse energy of the laser is 15.0 μJ, the grain size is the smallest. However, when laser single pulse energy remains 17.5 μJ or more, hydrogen evolution is serious, which affects the laser action and increases the pH of the cathode surface so that the promotion of the nucleation rate was reduced [2,4].

**Figure 7:**
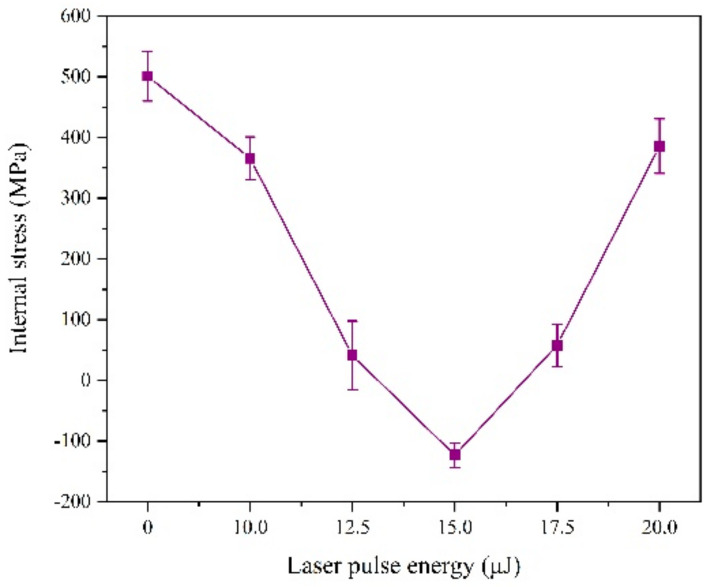
Internal stress of coatings with varying laser energies.

According to Figure 7, we find the laser irradiation can change the internal stress form of the coating from tensile stress to compressive stress.

This is because, on the one hand, part of the laser heat is absorbed by the reducing ions and exists as compressive stress after deposition. On the other hand, the laser has a force impact effect on the deposited layer. The joint effect of these two aspects can offset the tensile stress and even generate compressive stress during the electrodeposition process [15]. However, when the laser single pulse energy exceeds 15 μJ, the internal stress becomes the tensile stress again, the reason of which is that hydrogen evolution will intensify, affecting the laser action and density of the coating.

### 3.3. Micro-Hardness

**Figure 8:**
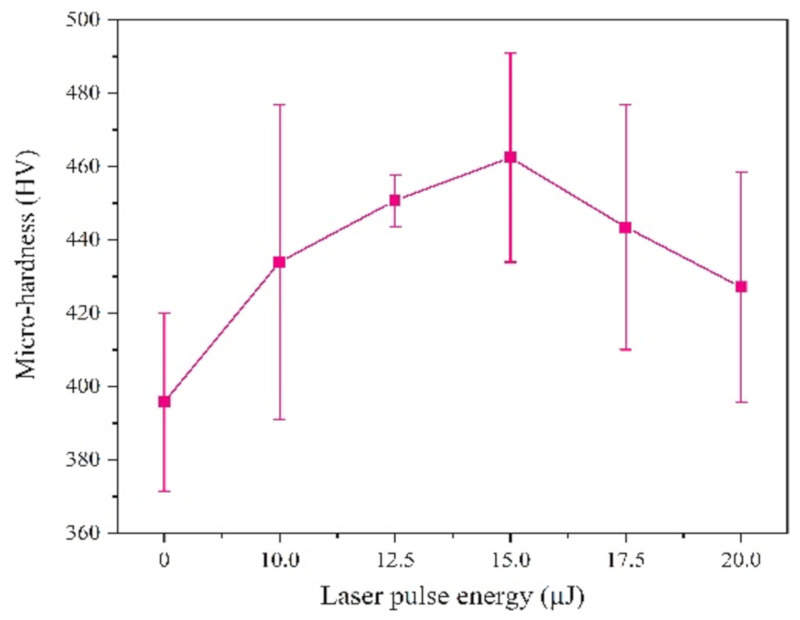
Micro-hardness of coatings with varying laser energies.

We tested the micro-hardness by selecting five points randomly, and the results shows that the micro-hardness with a laser is greater than that without laser, as can be seen from Figure 8, which is attributed to the grain size and chemical composition.

On the one hand, the effect of grain size on the micro-hardness is achieved by its effect on dislocation motion [22]. Laser irradiation will cause over-potential and the nucleation rate increasing, which leads to the coatings with smaller grain size. On the other hand, solid solution strengthening is an effective parameter on the micro-hardness of the coatings, which is mainly achieved by the influence of solute atoms on dislocation motion. The position of the Ni atoms was replaced by larger Fe atoms, causing lattice distortion and forming a stress field, which can strengthen the sediment layer [23]. The content of Fe is increased by laser irradiation, leading to the improvement of solid solution strengthening. When the laser single pulse energy is 15.0 μJ, the hardness of the coatings reaches maximum. However, when the laser single pulse energy is further increased, hydrogen evolution will reduce the structural density of the coating and generate large number of lattice defects, thereby reducing the micro-hardness.

### 3.4. Corrosion Behavior

**Figure 9:**
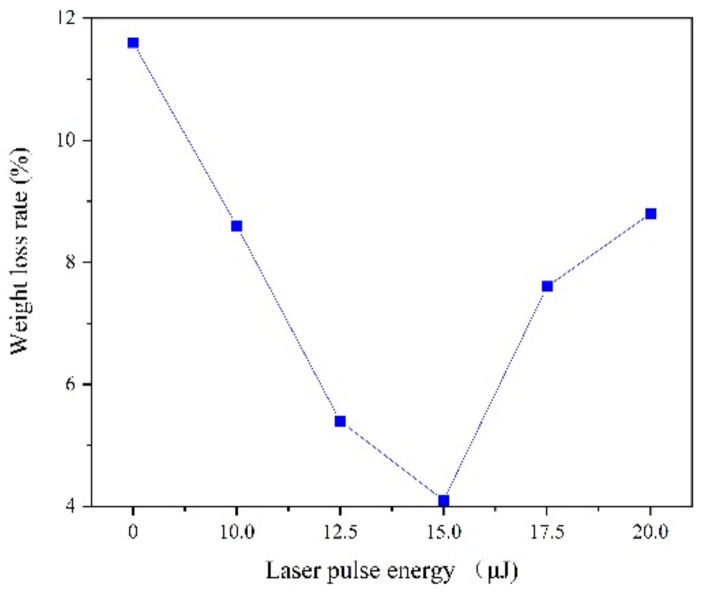
Corrosion behavior of coatings with varying laser energies.

The corrosion behavior was conducted in the 3.5% NaCl solution for seven days. When calculating the weight loss rate of the coating, a high-precision electronic balance (0.1 mg) is used to measure the quality of the coating before and after the deposition for 5 times. As shown in Figure 9 results indicate that the laser irradiation can reduce the weight of corrosion, which is related to the internal stress, grain size and surface morphology. In the 3.5% NaCl solution, hydrogen evolution corrosion occurred, which is characterized by easy dissolution of the Fe-Ni alloy. Cl^−^ can exacerbate corrosion, but grain size refinement hinders the penetration energy of Cl^−^ and OH^−^ plasma, which can reduce the soluble ions [24,25]. Another character is that large number of hydroxides and oxides (Fe_3_O_4_, Fe_2_O_3_, NiO, etc.) are formed due to the intensification of oxygen absorption corrosion, covering the surface of the electrode. Then different compositions of Fe-Ni alloy were passivated, which increases with the refinement of grain size, so the corrosion resistance is greatly improved [24,25,26].

Laser irradiation can promote grain size refinement, density of structure and improve the internal stress, which can increase the corrosion resistance. However, when the laser pulse energy is too large, the hydrogen evolution phenomenon is intensified, so that the coating structure generates lattice defects and the shortcomings emerge such as pits, pocking and cracks. Moreover, grain refinement is weakened and the penetration ability of the Cl^−^ and OH^−^ plasma is enhanced, so the corrosion resistance will be reduced.

### 3.5. Tensile Behavior

**Figure 10:**
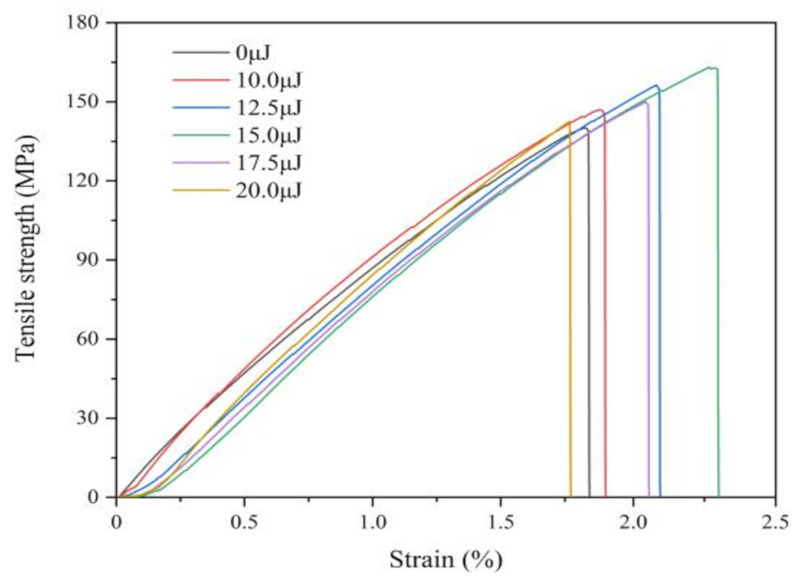
Tensile behavior of coatings with varying laser energies.

Figure 10 shows the stress-strain curves of coatings with varying laser energies, which is observed that the tensile property is significantly improved by laser irradiation. The tensile strength and elongation are 140.02 MPa and 83.55% without laser irradiation, while the laser pulse energy is 15.0 μJ, the tensile strength and elongation are 164.91 MPa and 172.97%, respectively. Because of laser irradiation, the grain size reduces and the number of grain boundaries increase, which make the generation of dislocations difficult. In addition, laser effect increases the Fe content and enhances solid solution strengthening. However, when the laser pulse energy is 17.5 μJ or more, the tensile strength gradually declines because of larger grain size and decreasing of Fe contents.

In summary, the chemical composition and grain size are the important factors in determining the mechanics performance of Fe-Ni alloy coating. The grain size is an effective parameter on the mechanics’ performance of the coating. The grain size was becoming smaller, the mechanics performance would be improved, and the difference in element content will change the grain structure in the coating and affect the mechanical properties of the coating [27]. Based on the observation and analysis, the appropriate laser parameter is helpful for the stability of laser enhanced electrochemical.

## 4. Conclusions

In this paper, a Fe-Ni alloy was prepared by constructing a laser electrochemical composite machining system by the manner of laser reciprocating scanning. The thermal and mechanical effects produced by the laser are beneficial to the electrodeposition process, which can increase the cathode over-potential and refine the grain. The laser effects on surface morphology, micro-structure, and mechanical properties were studied and results show that when the laser single pulse energy is 15 μJ, the enhancement effect on the coating morphology and performance is the best. This may be an effective and promising method for preparing other alloys.

## Figures and Tables

**Table 1:** Chemical composition of the electrolyte.

Parameters	Values
NiSO_4_·6H_2_O	120 g/L
FeSO_4_·7H_2_O	20 g/L
NiCl_2_·6H_2_O	40 g/L
H_3_BO_3_	40 g/L
Na_3_C_6_H_5_O_7_·2H_2_O	20 g/L
C_7_H_5_O_3_NS	3 g/L
C_12_H_25_SO_4_Na	2 g/L
Bath pH	3
Temperature	25 ℃
